# Addressing Methodological Concerns in PRP Treatment for Lichen Planopilaris: A Response to Willaert et al.

**DOI:** 10.1111/jocd.70180

**Published:** 2025-04-21

**Authors:** Abbas Dehghani, Zahra Lotfi, Azadeh Goodarzi

**Affiliations:** ^1^ Department of Dermatology, Rasool Akram Medical Complex Clinical Research Development Center (RCRDC), School of Medicine Iran University of Medical Sciences (IUMS) Tehran Iran

**Keywords:** lichen planopilaris, LPP, platelet rich plasma, PRP

Dear Editor,

We appreciate the opportunity to respond to the correspondence by Willaert et al., titled “Methodological considerations in PRP treatment for lichen planopilaris: addressing potential bias,” regarding our study on the efficacy of platelet‐rich plasma (PRP) in lichen planopilaris (LPP). We value their insights and acknowledge the importance of minimizing bias in clinical research.

While we recognize that the absence of double‐blinding may introduce a degree of performance bias, we would like to clarify several key points.

First, implementing a fully double‐blinded design in interventional studies involving PRP poses practical challenges. The procedure involves visible and perceptible differences between groups, making it difficult to effectively blind participants without the use of a placebo injection (e.g., saline). However, ethical concerns arise when administering an unnecessary scalp injection, which could potentially cause discomfort or introduce confounding variables.

Second, to mitigate bias, we incorporated objective assessments, including trichoscopic evaluations and photographic documentation, alongside patient‐reported outcomes such as the LPP Activity Index. The improvements observed in these objective measures reinforce the validity of our findings and suggest that the observed efficacy of PRP is not solely attributable to patient expectations.

Third, our control group received clobetasol, a well‐established treatment for LPP, rather than placebo. If performance bias had substantially influenced the results, we would expect a markedly exaggerated difference between groups. However, the data demonstrated a consistent and measurable benefit of PRP as an adjunct to clobetasol, highlighting its clinical relevance.

In conclusion, while performance bias is a recognized limitation in trials involving interventional procedures, we believe our study's design, inclusion of objective measures, and use of an active control group provide a robust assessment of PRP's efficacy. We appreciate the discussion on methodological refinements and look forward to future advancements in PRP research.

## Ethics Statement

The authors have nothing to report.

## Conflicts of Interest

The authors declare no conflicts of interest.

## Linked Articles

Methodological considerations in PRP treatment for lichen planopilaris: Addressing potential bias https://doi.org/10.1111/jocd.70157

## Data Availability

The authors have nothing to report.

